# Dynamic multimerization of Dab2–Myosin VI complexes regulates cargo processivity while minimizing cortical actin reorganization

**DOI:** 10.1074/jbc.RA120.012703

**Published:** 2021-01-07

**Authors:** Ashim Rai, Duha Vang, Michael Ritt, Sivaraj Sivaramakrishnan

**Affiliations:** Department of Genetics, Cell Biology, and Development, University of Minnesota Twin Cities, Minneapolis, Minnesota, USA

**Keywords:** Dab2, myosin VI, actin remodeling, motor processivity, cargo–motor interaction, AB, assay buffer, BG, benzyl-guanine, CBD, cargo-binding domain, FRAP, fluorescence recovery after photobleaching, MAC, minimal actin cortex, MIR, myosin interacting region, PS, phosphatidylserine

## Abstract

Myosin VI ensembles on endocytic cargo facilitate directed transport through a dense cortical actin network. Myosin VI is recruited to clathrin-coated endosomes *via* the cargo adaptor Dab2. Canonically, it has been assumed that the interactions between a motor and its cargo adaptor are stable. However, it has been demonstrated that the force generated by multiple stably attached motors disrupts local cytoskeletal architecture, potentially compromising transport. In this study, we demonstrate that dynamic multimerization of myosin VI-Dab2 complexes facilitates cargo processivity without significant reorganization of cortical actin networks. Specifically, we find that Dab2 myosin interacting region (MIR) binds myosin VI with a moderate affinity (184 nM) and single-molecule kinetic measurements demonstrate a high rate of turnover (1 s^−1^) of the Dab2 MIR–myosin VI interaction. Single-molecule motility shows that saturating Dab2-MIR concentration (2 μM) promotes myosin VI homodimerization and processivity with run lengths comparable with constitutive myosin VI dimers. Cargo-mimetic DNA origami scaffolds patterned with Dab2 MIR-myosin VI complexes are weakly processive, displaying sparse motility on single actin filaments and “stop-and-go” motion on a cellular actin network. On a minimal actin cortex assembled on lipid bilayers, unregulated processive movement by either constitutive myosin V or VI dimers results in actin remodeling and foci formation. In contrast, Dab2 MIR–myosin VI interactions preserve the integrity of a minimal cortical actin network. Taken together, our study demonstrates the importance of dynamic motor–cargo association in enabling cargo transportation without disrupting cytoskeletal organization.

Cytoskeletal molecular motors play essential roles in maintaining the subcellular architecture of eukaryotic cells. Through their engagement to distinct cargo adaptor proteins, they are targeted to distinct subcellular compartments (1, 2). Despite our detailed understanding of motor structure and the cargo adaptors that link motors to scaffolds, the structural and biochemical mechanisms that confer functional selectivity in diverse cellular contexts remain poorly understood. Functional selectivity of motor function emerges from the collective interactions between cargo complexes, motors, and the unique cytoskeletal architecture in distinct microenvironments inside the cell. This study focuses on adaptor proteins that bridge the interaction between motors and cargo and serve as the first point in cargo interface-mediated motor regulation. Initially thought to be passive connectors of motors to cargoes, adaptor proteins have since emerged as active modulators of motor structure and function. Studies focused on individual motor–cargo adaptor interactions have identified the release of autoinhibition (3), motor dimerization (4), and load-dependent changes upon cargo interaction (5), as common themes of structural regulation across cytoskeletal motors. However, it is unclear whether these established mechanisms provide sufficient modes of regulation to tune motor function inside cells. Additionally, the interplay between distinct modes of motor regulation that together confer functional selectivity remains an outstanding challenge.

The multifunctional, actin-based, molecular motor, myosin VI, displays functional selectivity in diverse cellular contexts (6). Cell biological observations postulate an anchoring role in stereocilia (7), tethering to the neighboring cytoskeleton for Golgi (8), and a transporter function in membrane traffic (9). This functional selectivity of myosin VI is enabled by the engagement of the motor to distinct subcellular compartments through diverse adaptor proteins (10). Myosin VI engages clathrin-coated endosomes through Dab2 (11), uncoated endosomes through GIPC (12), Golgi through optineurin (8), and also directly with PIP2 phospholipids (13). In addition to the cargo adaptors that directly interface with myosin VI, it is now apparent that the cargo–motor interaction can also be influenced by a network of interactions at the motor–cargo interface (14, 15). Additionally, the structural influence of distinct adaptors on myosin VI function remains an outstanding challenge. Specifically, myosin VI has a number of unique structural features, including a flexible extensible lever arm (16), cargo-mediated dimerization (17), release of an autoregulatory interaction (18), and load-sensitive changes to its chemomechanical cycle (19) that are selectively deployed to tune function. A precise understanding of how adaptors leverage these structural mechanisms to tune functional selectivity in cells remains unclear and is systematically dissected here.

In this study, we examine the regulation of myosin VI through the endocytic adaptor Dab2. The Dab2–myosin VI interaction occurs on clathrin-coated vesicles in the dense actin cortex near the plasma membrane (9). Early studies had defined the myosin VI interaction region (MIR) of Dab2 (11, 20). Subsequent studies examined the structural effect of Dab2 MIR binding on myosin VI (4, 17). In particular, using NMR and X-ray crystallographic structural analysis, Yu *et al.* showed that Dab2-MIR region comprises a 21 a.a. peptide ranging from a.a. 649 to 670 at the C terminus of Dab2 and consists of two α-helical motifs (defined as αA and αB in the paper) connected by a stalk. The αA and αB motifs bind distinct regions within a single myosin VI cargo-binding domain (CBD), resulting in a 2:2 Dab2-MIR-CBD complex with an apparent affinity of ∼0.5 μM. However, the functional implications of the Dab2 MIR:myosin VI CBD complex on myosin VI motility have not yet been elucidated and form the focus of this study.

Cytoskeletal motors are presumed to be stably bound to cargo during processive transport. However, multiple studies suggest the potential for motor–cargo turnover, given the weak Dab2–myosin VI interaction affinity (4, 21). Indeed, fluorescence recovery after photobleaching (FRAP)-based measurements of myosin VI and Dab2 on endosomes inside cells confirmed a high rate of motor turnover with a half-life of ∼13 s of myosin VI (22). The consequences of motor–cargo interaction turnover on motor function remain unclear and are dissected here using single-molecule motility, programmable synthetic cargos, cellular actin networks, and actin reorganization in a minimal actin cortex.

In this study, the MIR of Dab2 was first confirmed to be sufficient to dimerize the CBD of myosin VI. Single-molecule analysis of the binding kinetics of this interaction indicated a high turnover of the motor–cargo interaction, which is on par with the catalytic rate of the motor. Single-molecule motility assays suggested that adaptor-bound processive events are driven by motor dimers. Adaptor-bound motors were also found to be processive in surface actin gliding assays and contributed to processive ensemble movement of synthetic, programmable DNA nanostructure cargos. However, while this processive movement of cargo was found to have similar run lengths on a cellular actin network, the trajectories of adaptor-mediated cargo movement were found to have more pauses and to remain paused for longer. Interestingly, these features were found to combine to make Dab2-myosin VI motility less likely to destructively rearrange a minimal actin cortex into actin foci on supported lipid bilayers, as compared with constitutively dimerized and processive motors. Together, these findings indicate that Dab2 interactions with myosin VI are uniquely adapted to efficiently transport cargo through the dense cortical actin network at the periphery of the cell, without compromising actin network architecture.

## Results

### Dab2 binds and weakly dimerizes myosin VI through its cargo-binding domain

**Figure 1:**
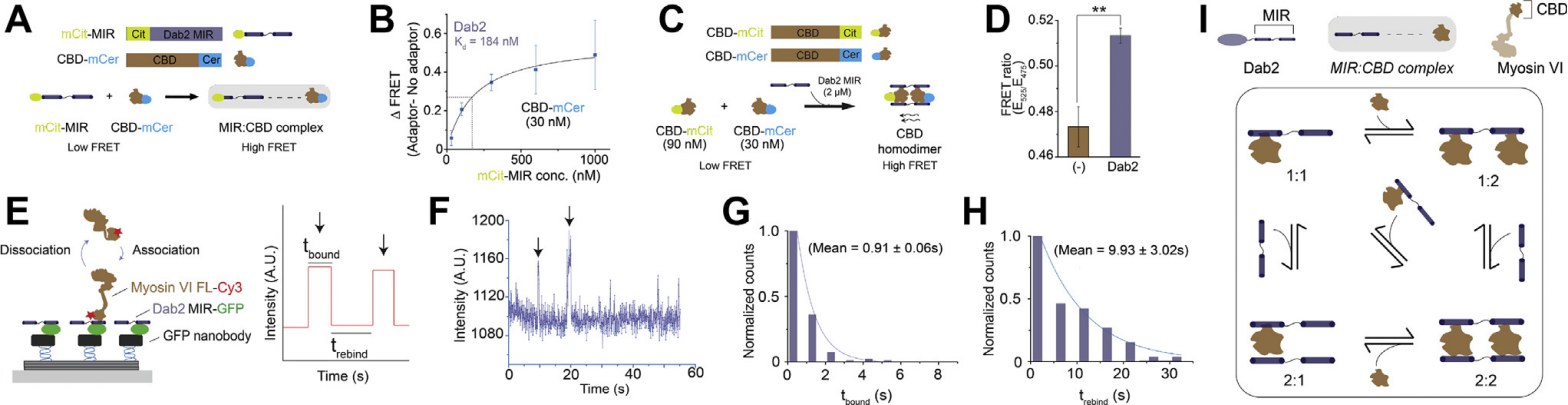
**Dynamic multimerization of myosin VI in the presence of the Dab2–myosin interacting region (MIR).** Dab2 dimerizes myosin VI through its cargo binding domain. *A*, schematic of the bimolecular FRET binding assay used to assess myosin VI dimerization through adaptor binding, forming the MIR:CBD complex. *B*, binding curve generated from the FRET binding assay done with increasing concentrations of mCit-MIR and a fixed concentration of CBD-mCer at 30 nM. *C*, schematic representing the dimerization FRET assay conducted using Dab2 MIR and labeled myosin VI CBD-mCer and CBD-mCit. *D*, saturating Dab2 (2 μM) concentrations enhance inter-CBD interactions (30 nM CBD-mCer + 90 nM CBD-mCit) as measured by FRET ratio (ratio of emission at 525 nm to the emission at 475 nm). *E*, schematic representation of the single-molecule TIRF binding assay used to measure adaptor–motor interaction kinetics (*left*) and the parameterization of mock data (*right*). *F*, representative intensity–time traces for single-molecule binding events of Dab2 to myosin VI. *Arrows* indicate observed events. *G*, distribution of the “bound” time for adaptor–myosin VI interaction (n = 75). *H*, distribution of the “rebinding” time for adaptor–myosin VI interaction (n = 34). *I*, schematic illustrating the possible interactions between the minimal myosin VI binding region (MIR) of Dab2 and cargo-binding domain (CBD) of myosin VI to form MIR:CBD complexes. Data are derived from three different preparations of the protein with three experimental replicates per preparation (mean ± S.D., n = 3). Error bars are S.D. Significance was computed using Student’s *t*-test (∗∗*p* ≤ 0.01).

The myosin VI binding interface for the endocytic adaptor proteins Dab2 has been well characterized (4, 22). To minimize regulatory effects arising from nonmotor interacting domains of the adaptor, we have used the minimal myosin VI interaction region (MIR) of Dab2 in this study. The Dab2 MIR has been demonstrated to dimerize myosin VI through a 2:2 interaction with its CBD (4). A FRET binding assay was conducted between fluorescently labeled Dab2 MIR and myosin VI to determine the concentrations at which saturable binding occurs, regardless of dimerization state (Fig. 1, *A*–*B*; K_d_ ∼ 184 nM). Future experiments used a saturating concentration of 2 μM (tenfold higher than K_d_) to drive 2:2 complex formation. Ratiometric FRET measurements using donor/acceptor-labeled myosin VI CBD showed that saturating concentrations of Dab2 MIR were sufficient to induce myosin VI CBD dimerization (Fig. 1, *C*–*D*).

Most experimental and theoretical studies of multimotor transport have been conducted by presuming the attachment of motors to cargoes as stable linkages that are maintained through the duration of cargo transport (23). However, FRAP measurements on cellular cargoes have shown that there is a turnover of motors on cargo inside cells (22). Therefore, it is essential to understand the kinetics of association and dissociation of the cargo–motor interaction. The binding kinetics of the adaptor–motor complex were probed using a single-molecule TIRF binding assay (Fig. 1*E*). Representative intensity-time traces show myosin VI binding events to single molecules of Dab2 tethered to a DNA nanotube (Fig. 1*F*). Analysis of the bound (t_bound_) and rebinding time (t_rebind_, the time between two sequential binding events at a single spot) revealed both a high turnover rate and a low rate of rebinding of the Dab2–myosin VI interaction (Fig. 1, *G*–*H*). The high rate of turnover observed (0.91 s^−1^) is on par with the reported acto-myosin ATPase cycle (∼1 s^−1^) (24), suggesting that on average only one ATPase cycle is likely to occur during the bound time of the adaptor. In summary, our data suggests that the Dab2 MIR/myosin VI interaction is dynamic, resulting in multiple possible states (Fig. 1*I*).

### Dab2 MIR effectively dimerizes and increases processivity of myosin VI at the single-molecule level

**Figure 2:**
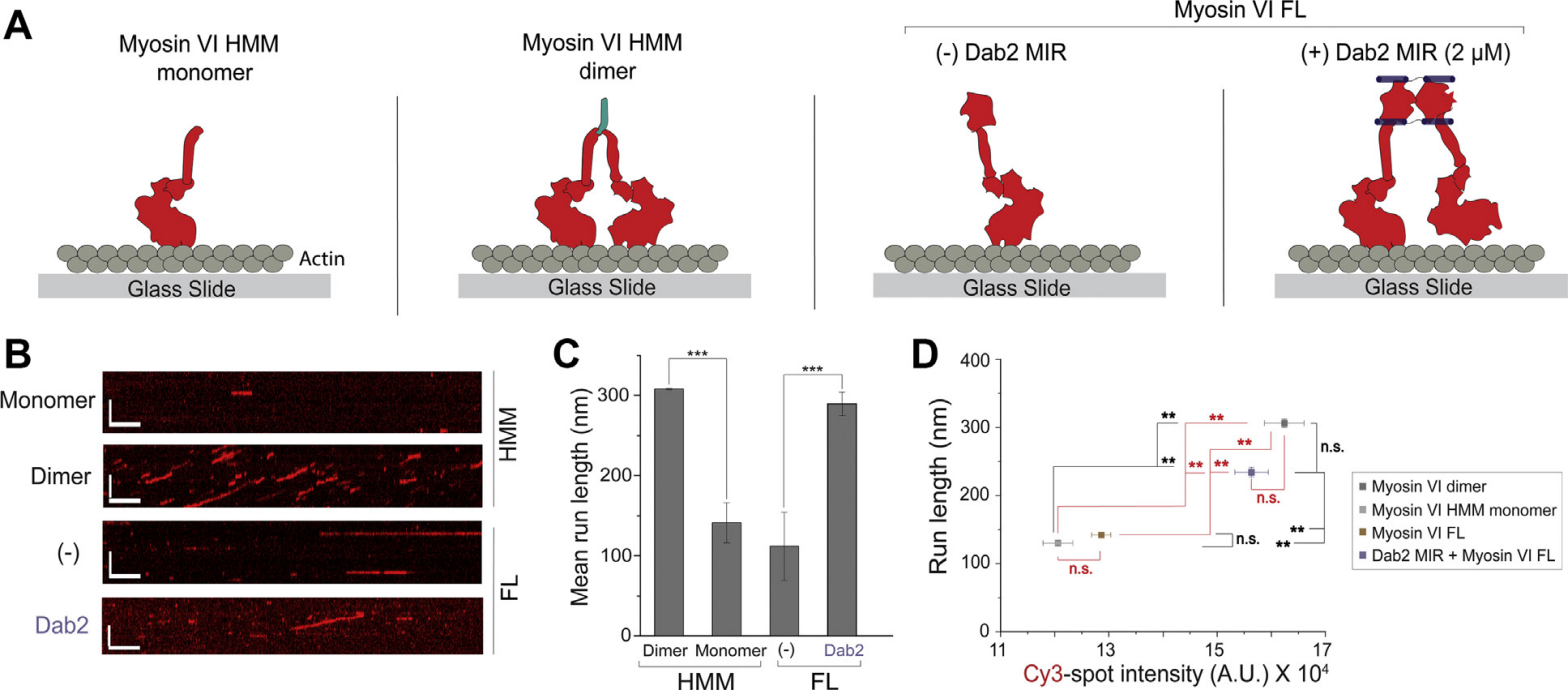
**Dimerization and processive movement of myosin VI by Dab2 at the single molecule level**. *A*, schematic illustrating the different motor arrangements observed in the single-molecule TIRF motility assay. *B*, representative kymographs depicting motility of a single adaptor–motor complex. Scale bars: vertical = 5 μm, horizontal = 50 s. *C*, estimation of the mean single-molecule run length from the TIRF motility assay. For each condition, more than 1000 motile events from three independent preparations of protein were used to estimate the mean run length. *D*, plot comparing run length and spot intensity of single molecule spots in a TIRF motility assay. Runs >500 nm were used to estimate run length and spot intensities. For each condition, spot intensities and run lengths were derived from 30 kymographs pooled from three independent preps of the proteins. Black significance bars indicate a comparison made for run length and red bars for spot intensity. Error bars are S.D. Significance was computed using Student’s *t*-test for panel *C* and a one way ANOVA with Tukey’s post-hoc test for panel *D* (∗∗*p* ≤ 0.01; ∗∗∗*p* ≤ 0.001; n.s., not significant).

The effect of Dab2 on myosin VI processivity was probed using single-molecule motility of the adaptor–motor complex (Fig. 2*A*). Complex formation between Dab2 MIR and myosin VI was assessed using two-color single-molecule imaging of GFP-tagged Dab2 MIR and Cy3-labeled full-length myosin VI (Fig. S1; Movies S1–S3). A GCN4 dimer of myosin VI HMM (HMM dimer) and a myosin VI HMM monomer (HMM monomer) were used to mimic processive and nonprocessive myosin VI motility respectively. The Dab2–myosin VI complex showed processive runs similar to the HMM dimer, while myosin VI alone displayed nonprocessive behavior akin to a monomer of myosin VI (Fig. 2, *B*–*C*). While previous studies have documented single-molecule processivity of myosin VI in the presence of Dab2, the dimerization state of the motor during motile events remains unclear. To assess this in our assay, the correlation of Cy3-labeled myosin VI spot intensity with run length during single-molecule motility events was used to determine the dimerization state of the motor. As expected, the Cy3-labeled HMM dimer had a spot intensity twice that of the HMM monomer and a significantly longer run length (Fig. 2*D*). Similarly, full-length myosin VI had a spot intensity and run length that was not significantly different from the HMM monomer (Fig. 2*D*). However, full-length myosin VI in the presence of Dab2 MIR was similar to the HMM dimer in terms of both run length and spot intensity (Fig. 2*D*).

### Dab2–myosin VI interactions result in ensemble processive motility

**Figure 3:**
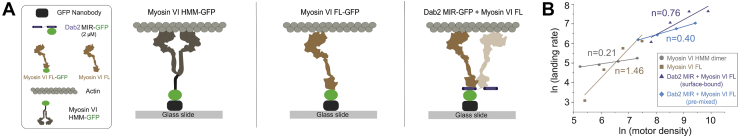
**Ensemble Dab2–myosin VI interactions enhance processive motility.***A*, schematic depiction of the surface actin gliding assay of myosin VI HMM-GFP, myosin VI-GFP, and dark myosin VI FL *via* a GFP-labeled Dab2 MIR. All GFP-labeled proteins are tethered to the surface through a nanobody specific for GFP. *B*, actin landing rate measurements at different myosin VI surface densities used to infer effect of adaptor binding on motor processivity. The slope of the linear fit was used to infer processive behavior, wherein processive motors have a slope ≤1, while nonprocessive motors have a slope ≥1. Each landing rate trace was computed from n > 500 actin landing events. The following were the R^2^ values of linear fit for each condition: myosin VI HMM dimer = 0.21, myosin VI FL = 1.46, surface-bound Dab2-myosin VI FL = 0.76, and premixed Dab2 + myosin VI FL = 0.40. All experimental conditions were performed across three different protein preparations (mean ± S.D., n = 3).

To further study the effect of Dab2 MIR binding on myosin VI motility, we reconstituted adaptor-mediated myosin VI motility using a surface actin gliding assay (Fig. 3*A*). Analysis of actin landing rates as a function of motor density on the surface is an established approach to infer motor processivity in surface motility assays (25). The slope of a linear fit to a logarithmic plot of landing rate *versus* motor density is used to interpret processive behavior in landing rate assays (26). Processive motors are characterized as having a slope ≤1. Constitutively dimerized myosin VI HMM was found to have a slope of 0.21, indicating a high degree of processivity (Fig. 3*B*). However, full-length myosin VI was found to have a slope of 1.46, demonstrating a lack of processivity. When Dab2 MIR was added, the slopes again dropped below one, regardless of whether the motor was patterned on the surface through surface-bound Dab2 MIR (0.76) or premixed with Dab2 MIR (0.40).

**Figure 4:**
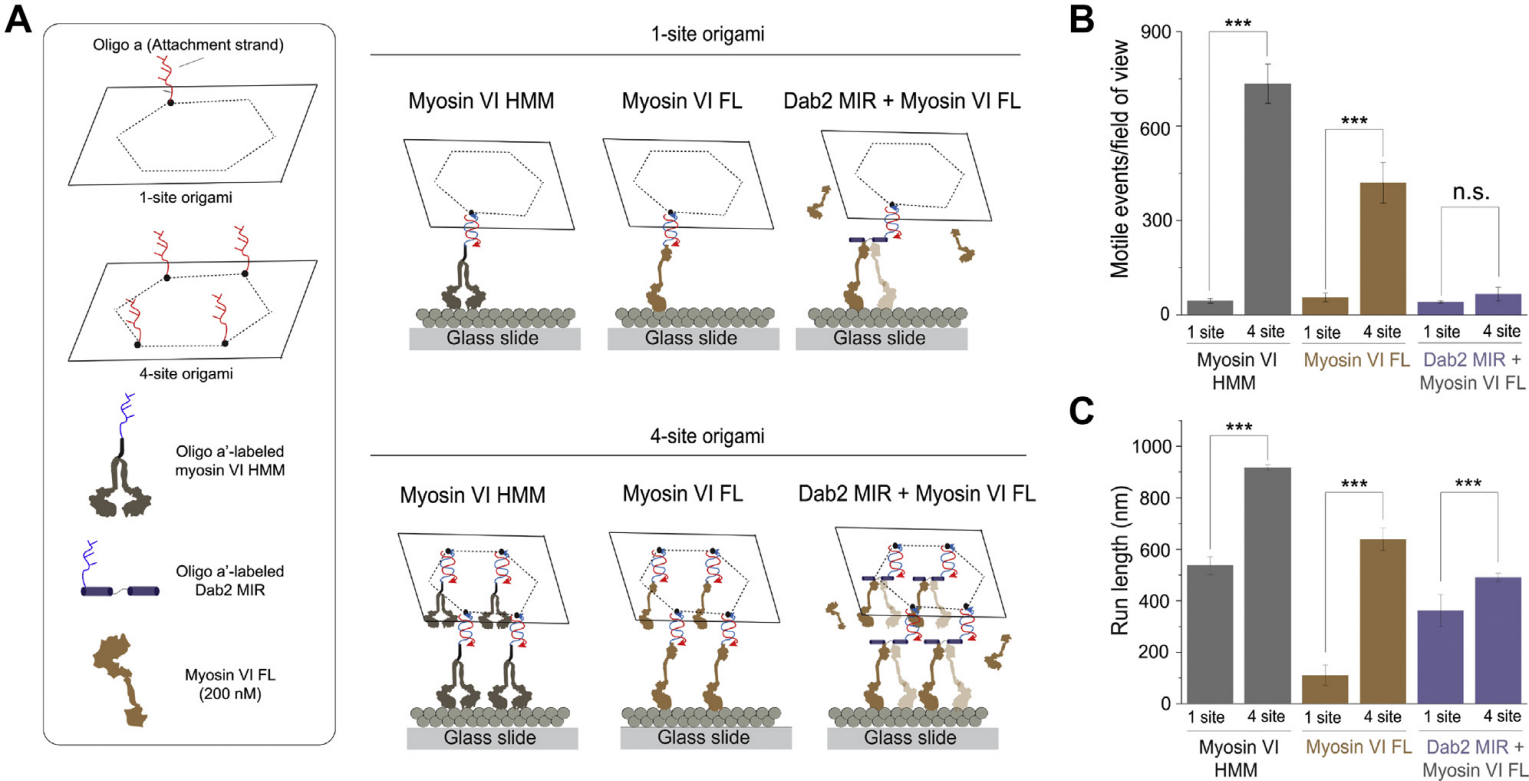
**Sparse processivity of multiple Dab2–myosin VI complexes on a synthetic cargo scaffold.***A*, schematic illustrating the geometry of one-site *versus* four-site DNA origami used for single actin filament motility assays of myosin VI HMM dimer, Dab2 MIR + myosin VI FL, and myosin VI FL. *B*–*C*, quantification of the number of DNA origami motile events per field of view (*B*) and run length (*C*) for the indicated conditions. Mean values depicted in *B*–*C* were estimated from ≥50 DNA origami motility events for each experimental repeat. All experimental conditions were performed across three independent protein preparations. Data shown in *B*–*C* represent the mean ± S.D. across preparations. Significance was assessed by Student’s *t*-test (∗∗∗*p* ≤ 0.001; n.s., not significant).

In order to understand the functional significance of ensemble processivity on collective myosin VI transport, we examined the motility of DNA nanostructures patterned with single (one-site) and multiple (four-site) cargo adaptors on single actin filaments (Fig. 4*A*) (27). We have previously demonstrated precise labeling of DNA nanostructures with predetermined numbers of proteins (27, 28). In agreement with the low binding affinity of Dab2 for myosin VI, we observed a significantly lower number of motile events with Dab2 as compared with HMM dimer, on both one-site and four-site nanostructures (Fig. 4*B*). The run length of Dab2 cargo movement significantly scales up from one-site to four-site nanostructures, similar to the constitutively processive HMM dimer (Fig. 4*C*). Together, these results demonstrate that Dab2 is able to drive sparse processive motility of myosin VI cargo at both single and ensemble motor levels.

### Adaptor interactions change the trajectory of myosin VI on cellular actin networks

**Figure 5:**
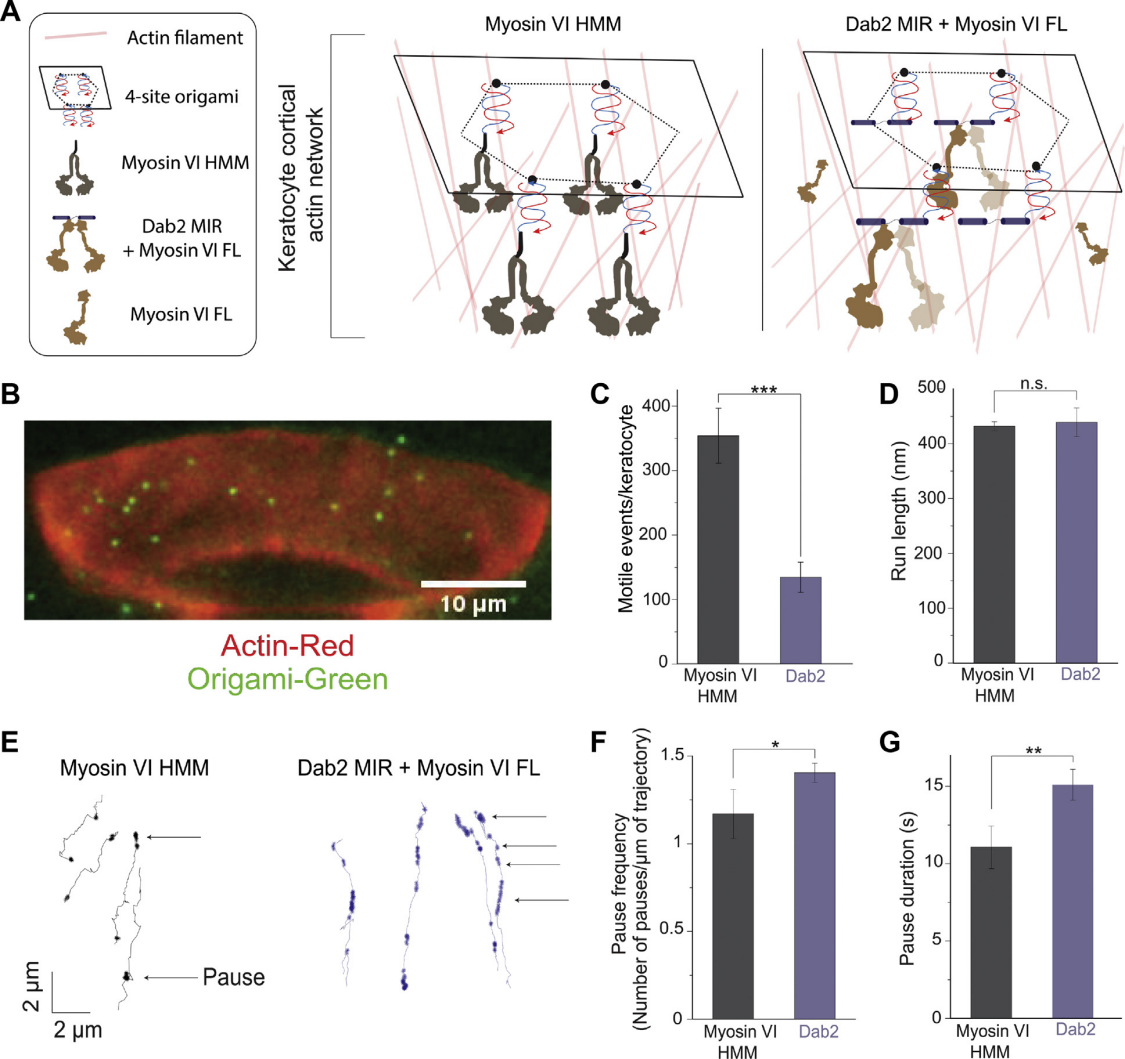
**Sparse “stop-and-go” movement of Dab2–myosin VI cargo complexes on a cellular actin network.***A*, schematic depicting four-site origami labeled with either myosin VI HMM or Dab2 MIR recruiting myosin VI FL on a keratocyte cortical actin network. *B*, representative image of an extracted keratocyte illustrating origami (*green*) on a cortical actin network (*red*). *C*–*D*, quantification of motile events (*C*) and run lengths (*D*) of each labeled origami condition. *E*, representative pauses in the trajectories of myosin VI HMM and Dab2-labeled origamis. *F*–*G*, quantification of pause frequency (*F*), and pause duration (*G*) of each labeled origami condition. All experimental conditions were performed across three independent protein preparations (mean ± S.D., n = 3). (∗*p* ≤ 0.05; ∗∗*p* ≤ 0.01; ∗∗∗*p* ≤ 0.001; n.s., not significant).

Using the same nanoarchitecture, we examined the effects of Dab2 MIR–myosin VI interactions in the context of cortical actin networks derived from fish-scale keratocytes (Fig. 5, *A*–*B*) (28, 29). Similar to observations made on single actin filaments, more motile events were observed with four-site origami labeled with myosin VI HMM dimers than with full-length myosin VI attached through Dab2 MIR (Fig. 5*C*). However, the observed run lengths of the two origami arrangements were similar, indicating the processive nature of Dab2-mediated motility (Fig. 5*D*). Interestingly, a pattern of more frequent pauses was observed in the trajectories of the Dab2 origami (Fig. 5, *E*–*F*). Additionally, these pauses were observed to last ∼1.5 times longer than with myosin VI HMM (Fig. 5*G*).

### Dynamic adaptor–motor interactions preserve actin organization in a minimal actin cortex

**Figure 6:**
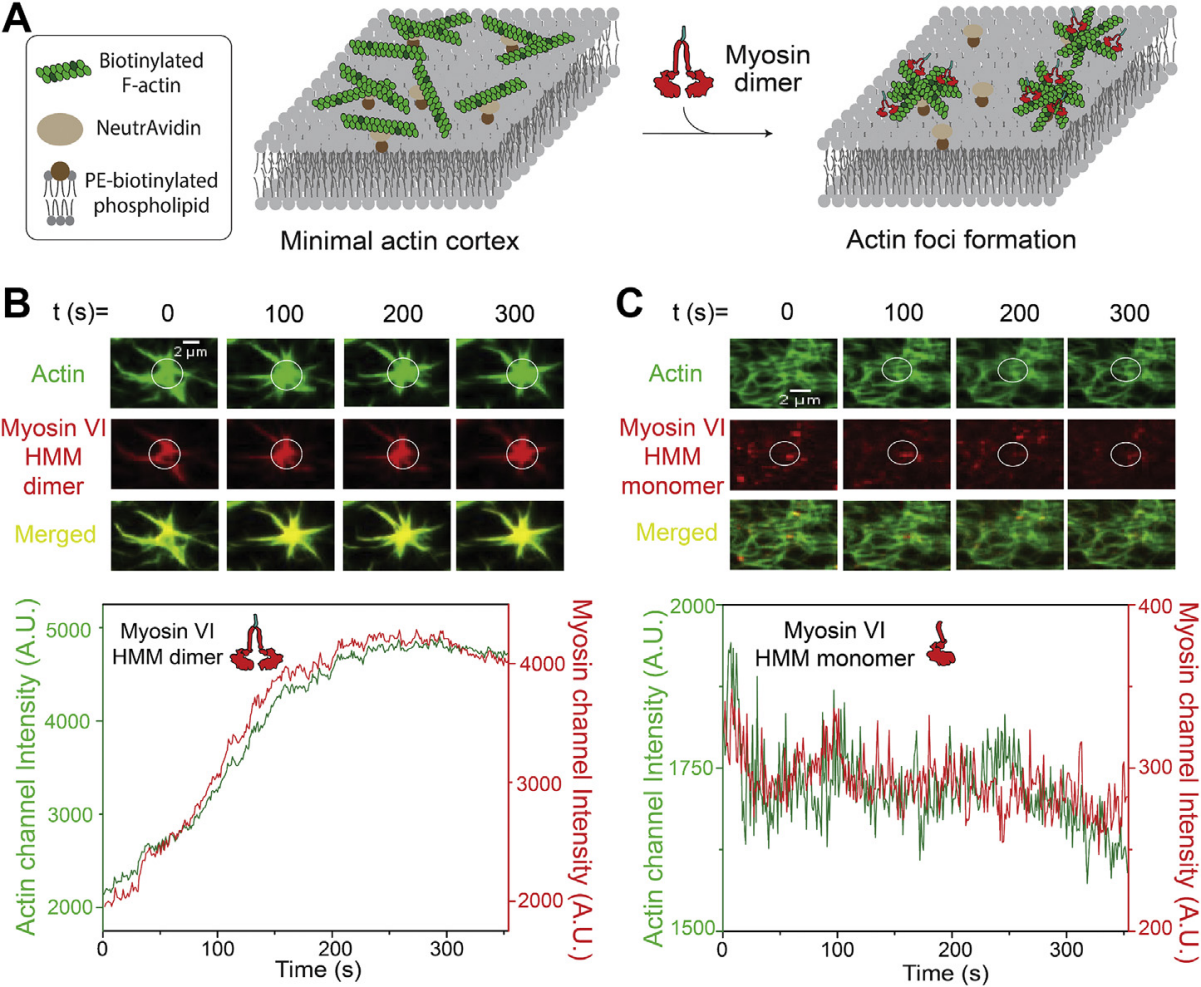
**Constitutive myosin VI dimers drive formation of actin foci on a minimal actin cortex.***A*, schematic representation of actin reorganization by the myosin dimer on a minimal actin cortex. *B*–*C*, representative time-lapse images from the dual-color imaging of the actin and myosin channels for myosin VI HMM dimer (*B*) and HMM monomer (*C*). Scale bar = 2 μm. The circle at the center of each image represents the region of the actin foci/network used for measuring intensity *versus* time. The corresponding intensity–time graphs for the actin and myosin channels are shown below each series of time-lapse images. All experimental conditions were performed across three independent protein preparations.

**Figure 7:**
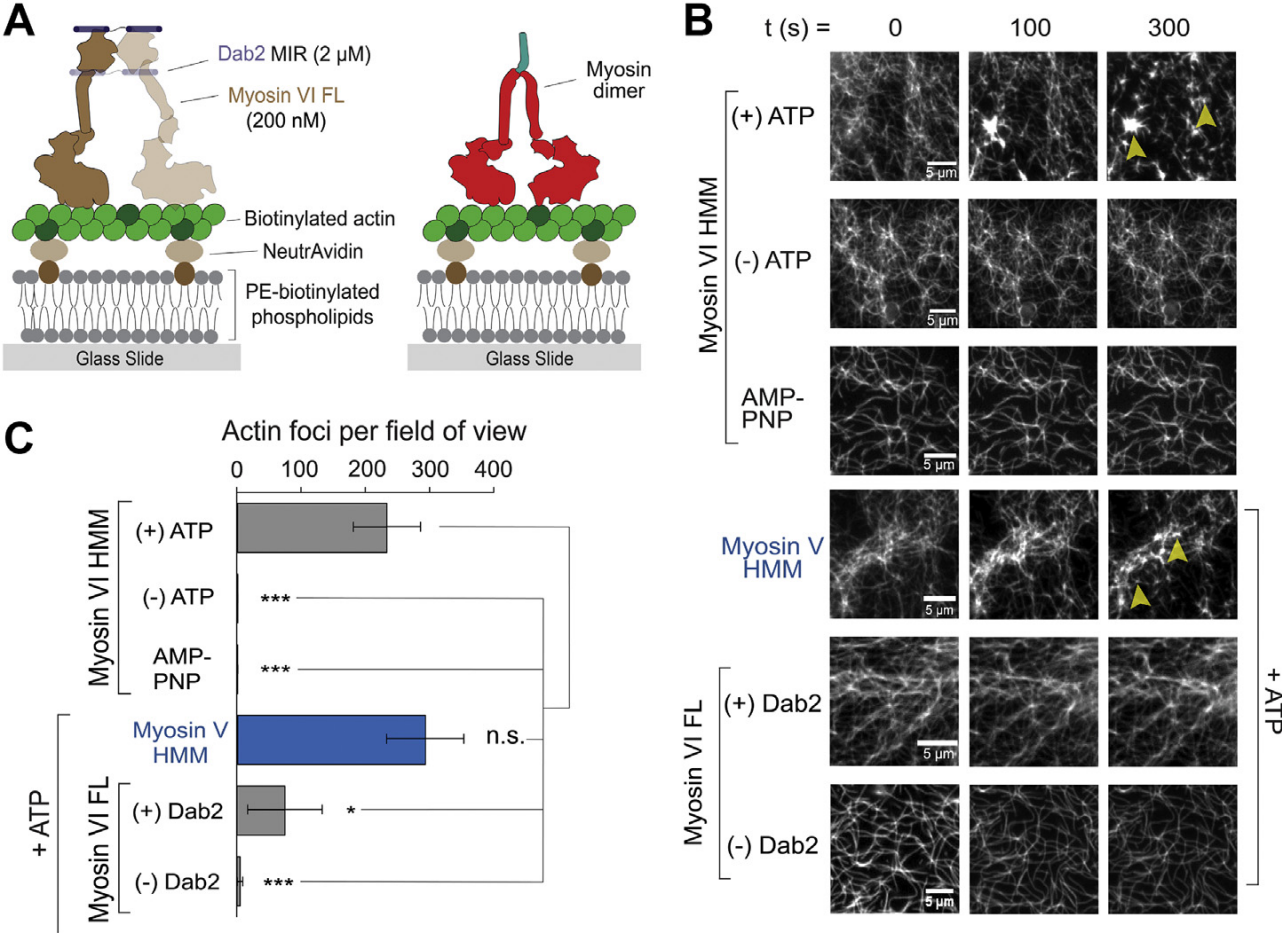
**Dynamic adaptor–motor interactions preserve actin organization in a minimal actin cortex.***A*, schematic summary of the actin remodeling assay. *B*, representative images of actin reorganization over time in the indicated motor and adaptor conditions. *C*, quantification of actin foci per field of view at 300 s for each condition. Data are derived from actin foci counts from three different images per three protein preparations (mean ± S.D., n = 3). Significance was computed using Student’s *t*-test (∗*p* ≤ 0.05; ∗∗∗*p* ≤ 0.001; n.s., not significant).

**Figure 8:**
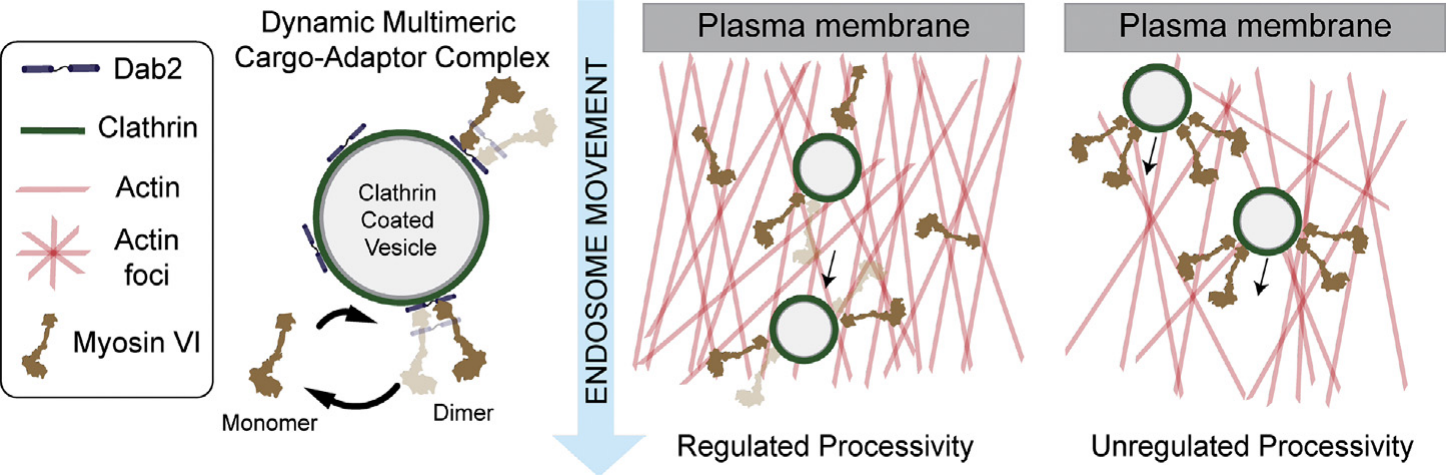
**Model for regulation of myosin VI processivity through Dab2 during endocytosis**.

Dab2 is present on clathrin-coated vesicles in the dense actin cortex near the plasma membrane (9, 30). Given the weak binding of Dab2 with myosin VI, we wanted to examine the effect of processive myosin VI movement on reorganization of a dense actin network. To mimic the cellular actin network, we assembled a minimal actin cortex (MAC) on a supported lipid bilayer to examine the effects of motor movement on actin organization (Fig. 6*A*) (31, 32). Constitutively dimerized HMM dimer of myosin VI causes a drastic reorganization of the MAC, resulting in the formation of actin foci, whereas monomeric myosin VI was unable to form actin foci (Fig. 6, *B*–*C*; Movies S4 and S5). To further understand the mechanistic basis of actin foci formation, we performed two-color imaging of actin and myosin VI in the actin remodeling assay. The HMM dimer but not the monomer displayed a centripetal accumulation toward the center of the actin foci (Fig. 6, *B*–*C*; Movies S6 and S7). The correlation between myosin accumulation and foci formation suggests that the actin remodeling of MAC is driven by dimeric, processive myosin VI. To expand these observations to Dab2, we performed the assays with full-length myosin VI and Dab2 MIR (Fig. 7*A*). Dimeric myosin VI HMM was able to cause the formation of actin foci only in the presence of ATP and not in its absence or the presence of a nonhydrolyzable ATP analogue (AMP-PNP, Fig. 7*B*). The requirement for ATP suggests that processive myosin VI movement mediates actin foci formation. The processive dimeric myosin V, which moves in the opposite direction as myosin VI (myosin V – plus-end; myosin VI – minus-end), was also able to generate actin foci (Fig. 7*B*). In contrast, full-length myosin VI was unable to form foci by itself (Fig. 7*B*). These findings suggest that a constitutive dimeric myosin is sufficient to drive actin foci formation. However, while in complex with Dab2 MIR, full-length myosin VI was observed to generate foci, although significantly fewer than observed with dimeric myosin VI (Fig. 7, *B*–*C*). Taken together, these data suggest that the dynamic interaction of myosin VI with Dab2 results in an equilibrium between monomeric and dimeric states of myosin VI that allows for processive motor movement on a dense actin network without significant remodeling of the underlying cytoskeletal network (Fig. 8).

## Discussion

In this study, we find that Dab2 binds weakly to myosin VI and facilitates dimerization at saturating concentrations, leading to sparse processivity. Despite the high turnover of the motor–adaptor interaction, Dab2–myosin VI ensembles retain sufficient acto–myosin interactions to maintain long-range processive movement on a cellular actin network. Interestingly, weak binding of Dab2 to myosin VI is necessary to minimize disruption of a minimal actin cortex resulting from the unregulated motor dimerization. Our findings suggest that dynamic multimerization of myosin VI by Dab2 tunes processive endosomal transport in cells.

Homo-dimerization of myosin VI has been a contentious issue (16), with initial studies identifying potential dimerization motifs within the full-length motor (33) and later studies confirming that full-length myosin VI was incapable of dimerizing on its own and needed cargo-assisted dimerization to function as a processive motor (34). Structural and functional studies showed that binding to PtdIns(4,5)P2 phospholipid or adaptor proteins such as Dab2 and optineurin could dimerize full-length myosin VI and make it processive (4, 13, 17). In agreement with the literature, we found that the adaptor, Dab2, was capable of dimerizing myosin VI using a bimolecular FRET-based assay (Fig. 1, *C*–*D*). Single-molecule and ensemble motility assays showed that dimeric myosin VI in complex with Dab2 moved processively on actin filaments (Figs. 2*C*, 3*B*, 4*C* and 5*D*).

The weak Dab2–myosin VI interaction, with off-rates (0.9 s^−1^; Fig. 1*G*) comparable with the acto-myosin ATPase cycle (1 s^−1^) suggests multiple possible interaction states within a Dab2–myosin VI ensemble (Fig. 1*I*). To examine the relative motility of these interaction states, individual Dab2–MIR monomers were constrained to interact with one or more myosin VI molecules in gliding filament assays (Fig. 3) and synthetic cargo on both single actin filaments and cellular actin networks (Figs. 4 and 5). These assays show that on single actin filaments, 1:1 or 1:2 Dab-MIR-myosin VI complexes are weakly processive with sparser and shorter runs compared with constitutive dimeric myosin VI. In contrast, driving 2:2 Dab2–MIR–myosin VI complexes using saturating adaptor concentrations results in run lengths comparable with constitutive dimers (Fig. 2*C*). Further, synthetic cargo with 1:1 and 1:2 complexes shows long-range processive movement (Figs. 4*C* and 5*D*) with a distinct “stop-and-go” behavior on cellular actin networks (Fig. 5, *E*–*G*). Taken together, these data suggest that dynamic multimers of Dab2–myosin VI are compatible with processive cargo transport.

While homo-dimerization of myosin VI by Dab2 is consistent with the need for motor processivity to bring about directed transport through the actin cortex, persistent interactions involving multiple dimeric motors could remodel the surrounding actin network to impede vesicle traffic. Indeed, multiple studies with dimeric myosin II motors on model supported bilayers have demonstrated significant reorganization of actin networks through myosin activity (35, 36). Independently, Reymann *et al.* (37) showed that myosin VI and myosin II organized on micropatterns were capable of reorganizing actin networks. In this study, we demonstrate that unregulated processive movement by a constitutively dimeric myosin VI can cause significant remodeling of model cortical actin networks assembled on supported lipid bilayers (Figs. 6 and 7). Actin foci formation is a hallmark of constitute motor dimerization as witnessed by equivalent effects for myosin V and VI HMM dimers (Fig. 7*C*). Simultaneous imaging of actin and myosin VI during actin aster formation shows that processive myosin VI movement generates a directed force leading to centripetal accumulation of actomyosin and actin foci formation (Fig. 6, *B*–*C*). The requirement for motor activity is also established from the lack of actin reorganization in the absence of nucleotide, the presence of ADP or AMP-PNP, a nonhydrolyzable ATP analog (Fig. 7, *B*–*C*). In contrast, nonprocessive constitutively monomeric myosin VI molecules are unable to coordinate their acto–myosin interactions to disrupt actin organization (Fig. 6*C*). Taken together, these data indicate that regulation of processive movement might be necessary for efficient myosin VI cargo transport through a dense cortical actin network without significant remodeling of the actin cytoskeleton.

Theoretical and experimental studies of motor proteins have assumed motors to be attached to the cargoes as stable, static linkages during the course of cargo transport (23). In contrast, we find that Dab2 interacts weakly with myosin VI, leading to sparse processive events. Increasing the number of motor–cargo complexes does not substantially enhance the number of processive events compared with constitutive dimers (Fig. 4*C*). Even at saturating adaptor concentrations that promote formation of 2:2 Dab2–myosin VI complexes, sparse processivity minimized the reorganization of a minimal cortical actin network (Fig. 7, *B*–*C*). These observations suggest that weak motor–cargo interactions are necessary to minimize disruption of the local actin network. Our *in vitro* measurements of a weak Dab2–myosin VI interaction are consistent with the turnover of the myosin VI on clathrin endosomes observed in live cell FRAP experiments (22). The greater half-life of the cargo interaction in cells (∼12 s) compared with single-molecule measurements (∼1.2 s) likely stems from the multivalent interaction of myosin VI with Dab2, PIP2, and clathrin (11, 13). Regardless of the interaction lifetimes, our study highlights the importance of cargo–adaptor turnover in tuning the functional selectivity of cytoskeletal motors.

Recent studies across different classes of cytoskeletal motors highlight the push to understand multiplexed regulation of motors as the next exciting frontier in cytoskeletal transport research. For instance, the dynein transport complex comprises multiple regulators such as Lis1–NudE complex, BICD2, the Hook family of proteins, and dynactin (38). Lis1-NudE provides load-dependent anchoring (5, 39), whereas BICD2 and Hook enhance processivity by enhancing the dynein–dynactin interaction (40, 41). In parallel, kinesin interaction with JIP1 triggered by MAPK phosphorylation on neuronal vesicles releases motor autoinhibition (42, 43). A well-characterized example of multiplexed regulation of myosin motors is the Rab27a–melanophilin complex, wherein melanophilin simultaneously bridges the cargo interaction with the actin track and releases myosin V autoinhibition to enhance processivity (44, 45). In addition to cargo adaptors, cytoskeletal motors can directly interface with cargo membranes through selective interactions with signaling lipids such as phosphoinositides and membrane-organizing lipids such as cholesterol. Lipid interactions have been shown to impact motor activation, processivity, and load-dependent behavior for myosin I, kinesin 3, and dynein (46, 47, 48). Given such a diversity of regulatory mechanisms, our study highlights the need to understand how they are multiplexed to provide functionally selective regulation. Specifically, for Dab2, the combination of weak binding and homo-dimerization enhances processivity without compromising the actin network during clathrin-mediated endocytosis. (Fig. 8).

Our study builds a strong mechanistic foundation for multiplexed regulation of myosin VI using the isolated myosin interacting regions of cargo adaptors. However, adaptors can function within protein interaction networks that could modulate motor regulation (15). In the context of the current study, in addition to Dab2, myosin VI has been shown to directly interact with clathrin and PIP2 on clathrin-coated vesicles (13, 49). The effect of this multivalent interaction network on myosin VI function on the same cellular structure needs to be explored in future studies. Further, the adaptor protein might be regulated by upstream binding to membrane receptors on endocytic vesicles. Dab2 has been shown to interact directly with the C-terminal tail of receptors such as the ion transport receptor megalin in the proximal tubule (50, 51). Hence, future efforts are necessary to dissect the role of upstream signals in the dual regulation of adaptor and motor.

## Experimental procedures

### List of constructs

All constructs were cloned into pBiex1 (Novagen) with C-terminal FLAG tags for purification unless otherwise noted. Dab2 MIR: a.a. 675 to 713 of human Dab2 was used as the myosin VI interaction region as defined previously (4). This construct was expressed with either a C-terminal fluorophore (eGFP, mCerulean, or mCitrine as indicated) connected through a flexible (Gly-Ser-Gly) x 2 repeat or without a fluorophore (dark).

GFP nanobody: GFP nanobody construct (52, 53) with a C-terminal SNAP tag with FLAG and 6x-His tags.

Myosin VI FL: The isoform of human myosin VI containing both the long insert (LI) and short insert (SI) was used as the starting point for all of the following constructs used in this study:

1. Myosin VI FL-GFP: Myosin VI with a C-terminal GFP tag
2. Myosin VI FL-FLAG: Myosin VI without a fluorescent tag.
3. Myosin VI FL-SNAP: Myosin VI with a C-terminal SNAP, FLAG, and 6X-His tags
4. Myosin VI HMM dimer: Myosin VI, residues 1 to 992 from *Sus scrofa*, containing both the IQ and SAH domains with a GCN4 leucine zipper (for dimerization), and SNAP, FLAG, and 6X-His tags.
5. Myosin VI HMM monomer: same as HMM dimer, but lacking the GCN4 leucine zipper.
6. Myosin VI CBD constructs: The C-terminal a.a. 1030 to 1284 of human myosin VI was used as the putative CBD of myosin VI as defined previously (4). Tagged with either mCitrine or mCerulean.

Myosin V HMM dimer: Myosin Va residues 1 to 1103 from *Gallus gallus* followed by a GCN4 leucine zipper (for dimerization), and SNAP, FLAG, and 6X-His tags.

The following previously reported and characterized extinction coefficients were used:

eGFP: 55,900 cm^−1^ M^−1^

mCerulean: 43,000 cm^−1^ M^−1^

mCitrine; 77,000 cm^−1^ M^−1^

Cy3: 150,000 cm^−1^ M^−1^

### Buffers and reagents

Assay buffer (AB): 20 mM Imidazole (pH 7.5), 25 mM KCl, 4 mM MgCl_2_, 1 mM EGTA, 1 mM DTT.AB.BSA: AB containing 1 mg/ml bovine serum albumin (BSA). AB.BSA.Cam: AB.BSA containing 10 μM calmodulin. AB.BSA.nt: AB.BSA containing 0.1 μM random nucleotide mix. Lysis Buffer (for protein purification): 20 mM Imidazole (pH 7.5), 200 mM NaCl, 4 mM MgCl_2_, 0.5 mM EDTA, 1 mM EGTA, 0.5% IGEPAL, 7% sucrose. Wash Buffer (for protein purification): 20 mM Imidazole (pH 7.5), 150 mM KCl, 5 mM MgCl_2_, 1 mM EDTA, 1 mM EGTA.

### Protein expression and purification

All proteins used in this study were expressed and purified in Sf9 insect cells and purified using a FLAG-tag-based affinity purification. The protocol is described briefly here. Transient transfection of constructs in Sf9 cells was achieved using the Escort IV system (MilliporeSigma). For protein purification, transiently transfected Sf9 cells at a cell number of ∼60 × 10^6^ cells were centrifuged at 350*g* for 5 min to pellet the cells. The supernatant was discarded and the cell pellet was resuspended in 3 ml of ice-cold lysis buffer supplemented with 1 μg/ml PMSF, 10 μg/ml aprotinin, and 10 μg/ml leupeptin. Cell lysis was achieved by 20 cycles of pipetting of the resuspended cell pellet. The cell lysate was centrifuged at 176,000*g* for 25 min at 4 °C in a TLA 100.4 rotor (Beckman Coulter) to pellet the cell debris. The supernatant was incubated with 50 μl of anti-FLAG M2 affinity resin for 2 h at 4 °C with rotation. The resin–lysate mix was centrifuged at 1000*g* for 1 min at 4 °C to pellet the resin. The resin was then washed three times with ice-cold wash buffer supplemented with 1 μg/ml PMSF, 10 μg/ml aprotinin, and 10 μg/ml leupeptin by resuspending and then pelleting the resin by centrifuging at 1000*g* for 1 min at 4 °C. The supernatant from the last wash was removed, and the resin was resuspended in wash buffer supplemented with 0.2 mg/ml FLAG peptide (MilliporeSigma) to elute the protein. Protein estimation was done either using a NanoDrop spectrophotometer (ThermoFisher) for fluorescently tagged proteins or using BSA standards on a 10% SDS-PAGE gel for nonfluorescent proteins.

### Conjugation of oligo with benzyl-guanine (BG) ester

Oligo with a 5’ amino modification (AmMC6, IDT) at a final concentration of 168 μM was mixed with benzyl-guanine NHS ester (NEB) at a concentration of 11.6 mM in 100 mM sodium borate buffer (pH 8.5) and incubated at 37 °C for 4 h with rotation. Labeled oligo was then purified using Illustra G-50 micro columns (GE Healthcare) twice, and the concentration of oligo was estimated using a NanoDrop spectrophotometer (ThermoFisher).

### Oligo labeling of SNAP-tagged proteins for DNA scaffold assays

Protein purification was carried out as described above. However, instead of eluting, proteins were incubated with oligos as described below. The resin was resuspended in 200 μl of wash buffer, and BG-labeled oligo was added at a final concentration of 1.5 μM. The labeling reaction was then incubated at 4 °C overnight with rotation. Postincubation, the resin was washed three times with wash buffer to remove the excess oligo, and elution using FLAG peptide was carried out as described above.

### Surface actin gliding motility assay

Plasma cleaned glass coverslips (22 × 22 mm, Corning) were coated with 0.1% colloidin (EMS) in amyl acetate. Flow chambers were prepared by sticking the colloidin-coated coverslips to a glass slide using strips of double-sided tape. GFP nanobody was added to the flow chamber at a final concentration of 200 nM in AB for nonspecific surface adsorption by incubation for 4 min at room temperature. The unbound GFP nanobody was washed off with three washes of AB. Surface passivation was then carried out by AB.BSA incubation for 4 min. Then, the GFP-tagged protein myosin VI FL, at a concentration of 200 nM, was added to the chamber and incubated for 4 min. The unbound protein was washed off by three washes with AB.BSA. For adaptor MIR-based assays, Dab2 MIR was premixed with myosin VI FL-FLAG and incubated for 5 min before flowing into the chamber and incubating for 4 min. The unbound myosin VI was washed with AB.BSA. Finally, a motility mix was prepared in AB.BSA.Cam buffer containing Alexa-647 phalloidin (Invitrogen) labeled F-actin at 0.5 μM, an ATP regenerating mix (1 mM phosphocreatine and 0.1 mg/ml creatine phosphokinase), an oxygen-scavenging system (0.6% glucose and 45 μg/ml catalase, 25 μg/ml glucose oxidase), and 2 mM ATP and added to the flow chamber. The actin gliding motility was assayed using a Nikon Eclipse Ti inverted epifluorescence microscope at 100× using a 1.4 NA oil-immersion objective at a frame rate of 1 Hz for 2 min using Nikon Elements software (Nikon). The actin gliding data was analyzed using FIESTA software (54). For actin gliding motility experiments, the myosin VI concentration was serially diluted starting from 200 nM to the lowest dilution at which actin landing on the surface could no longer be observed. When adding the Dab2 MIR, the adaptor protein was kept at 1 μM and mixed with a serial dilution of myosin VI starting from 800 nM to 25 nM. Surface density of motors and actin landing rates were calculated as described previously (26).

### DNA scaffold preparation and labeling

DNA nanostructures were prepared based on the detailed description in our previous work (27, 28). Briefly, single-stranded M13mp18 DNA (NEB) was mixed with fourfold excess of staple strands (IDT), followed by a 2 h annealing procedure. Intact scaffolds were separated from excess staple strands using Amicon Ultra 100K cutoff spin columns (MilliporeSigma). Purified scaffolds were mixed with 200 nM of oligo-conjugated SNAP-tagged protein, a mixture of 42-nucleotide oligos with randomized sequences (blocking oligos), and 1 to 5 μM calmodulin in 1× AB.BSA. After 10 min of incubation at room temperature, excess streptavidin-coated magnetic beads (NEB) were added and incubated at room temperature with shaking for 10 min. The beads were washed with AB.BSA.CAM. Finally, the beads were incubated in AB.BSA.CAM containing an imaging solution of 2 mM ATP, 1 mM phosphocreatine, 0.1 mg/ml creatine-phosphokinase, 45 μg/ml catalase, 25 μg/ml glucose oxidase, 1 to 2% glucose, and an excess of an elution strand for removal of origami from the beads using branch migration. For the Dab2 Mir condition, myosin VI at 1 μM concentration was also added in this step.

### Single-molecule TIRF motility assay

Flow chambers were prepared as outlined above for surface and nanotube motility assays. BSA conjugated with biotin was added to a flow chamber at 1 mg/ml in AB and incubated for 4 min. Unbound BSA-biotin was removed with three flows of AB and AB.BSA was added to the chamber for surface passivation for 4 min. Neutravidin at 0.2 mg/ml in AB.BSA was added to the flow chamber and incubated for 4 min. Unbound neutravidin was removed with three flows of AB.BSA. Biotinylated F-actin (1:9 ratio of biotin-G-actin: G-actin) was then added to the flow chamber at a concentration of 0.5 μM and incubated for 4 min. Unbound actin filaments were washed off with three flows of AB.BSA. The Dab2-MIR-GFP + Cy3-myosin VI complex or Cy3-myosin VI alone was added to the flow chamber at a concentration of 50 pM in a motility mix similar to that used for the surface motility assay. Dual-color imaging of single-molecule motility was done on a Zeiss TIRF microscope equipped with a Coherent 100 mW 488 nm and 561 nm OPSL laser and a 100× 1.4 NA oil-immersion objective with a DualView 2 for simultaneous two-channel imaging and a Photometrics QuantEM 512SC EMCCD camera for high-sensitivity single-molecule detection. Images were acquired using ZEN imaging software (Zeiss). Single-molecule motility was assayed at a frame rate of 10 Hz. Particle tracking of single-molecule motility data was done using the Trackmate (55) plugin in FIJI (56, 57). Kymographs of single-molecule motility were generated using the reslice tool in FIJI. Spot intensities of Cy3-myosin VI were obtained by a line intensity scan along the kymograph in the Cy3 channel. Run lengths of adaptor–myosin VI complexes were obtained from the analysis of kymographs of motile runs in the adaptor MIR-GFP channel.

### Bimolecular FRET assay for measuring K_d_ of adaptor–motor interaction

The CBD of myosin VI (991–1294) was expressed with a c-terminal mCerulean (CBD-mCer). DAB2-MIR was expressed with a c-terminal mCitrine. CBD-mCer was held constant at 30 nM and increasing concentrations of DAB2-MIR-mCit were titrated in. Fluorescent spectra were collected using an excitation of 430 nm (bandpass 4 nm) and emission from 450 to 650 nm (bandpass 2 nm). To account for cross-excitation of mCitrine, additional spectra were collected at the same concentration of the respective MIR-mCitrine without CBD-mCer. These cross-excitation spectra were then subtracted from the original spectra. Every spectrum was collected three times from three separate preparations of protein. Data was collated and fit using Origin (Oracle).

### Bimolecular FRET assay for myosin VI dimerization

A reaction mix for bimolecular FRET measurement was prepared in AB.BSA.Cam buffer comprising 30 nM of the CBD-mCer (FRET donor), 90 nM CBD-mCit (FRET acceptor), 2 mM ATP, and saturating concentration of adaptor at 2 μM. FRET measurements were performed on a Fluoromax-4 spectrofluorometer (Horiba Scientific) by exciting protein samples at 430 nm (mCer) with an 8-nm band pass, and emission monitored from 450 to 650 nm. The FRET ratio was calculated from the ratio of the emission for mCit (525 nm) to mCer (475 nm). For each experimental condition, three independent protein batches were used, and two replicates were measured for each independent experiment. Cross-excitation of the acceptor at donor excitation wavelengths was not found to contribute significantly to FRET at the concentrations of protein used in this assay. While there is contribution to the acceptor emission signal from the donor emission, we have not subtracted the donor emission before calculating the FRET ratio. We have previously shown that this ratio normalizes the contribution of the donor at acceptor fluorescence (58).

### Single-molecule TIRF binding assay

The detailed protocol for preparing DNA nanotubes has been described by previously (59). DNA nanotubes were patterned on the glass coverslip surface in flow chambers similar to that described above. GFP nanobody-SNAP was conjugated to an oligo and was then added to the chamber at a concentration of 200 nM in AB.BSA and incubated for 5 min to pattern GFP nanobody on the nanotube surface. Unbound GFP nanobody was removed with three washes with AB.BSA. Adaptor–GFP fusion proteins were then added at a concentration of 200 nM to the chamber to bind to GFP nanobody on the nanotube. Unbound adaptor-GFP was removed with three washes with AB.BSA. Finally, Cy3-labeled myosin VI was added to the chamber at a concentration of 50 pM in AB.BSA.Cam supplement with an oxygen-scavenging system (0.6% glucose and 45 μg/ml catalase, 25 μg/ml glucose oxidase). Single-molecule binding was assayed on a Zeiss TIRF microscope equipped with a Coherent 100 mW 488 nm and 561 nm OPSL laser and a 100× 1.4 NA oil-immersion objective with a DualView 2 for simultaneous two-channel imaging and a Photometrics QuantEM 512SC EMCCD camera for high-sensitivity single-molecule detection. Images were acquired using ZEN imaging software (Zeiss). Single-molecule binding was assayed at an acquisition frame rate of 20 Hz. Each assay was done by taking an image in the GFP channel to obtain a surface map of adaptor-GFP localization on the DNA nanotube. Then, the imaging was switched to the Cy3 channel and the Cy3-myosin VI binding assayed at an acquisition rate of 20 Hz for 3 min. Data analysis for single-molecule binding was done using FIJI (56, 57). First, the overlay of the GFP channel image was performed on the Cy3 channel movie to obtain a surface localization map of adaptor molecule at which the myosin VI binding occurred. Intensity *versus* time traces were then obtained at single spots using the time series analyzer plugin in FIJI. The intensity–time data was then plotted in Origin to calculate the bound and rebound time values from the traces. The distribution of binding and rebinding times was fitted to a single-exponential decay in Origin to obtain the mean values. The CDF for bound and rebind times was plotted using a custom-written code in Matlab. For the photobleaching control experiment for Cy3-myosin VI, nanotube assembly was done as described above and oligo-conjugated Cy3-myosin VI was added at a concentration of 50 pM to obtain single tethered spots of Cy3-myosin VI on DNA nanotubes. The spots were imaged and intensity–time data was plotted as described above.

### DNA origami motility on keratocyte actin network

Keratocytes were derived from the scales of *Thorichthys meeki* (Firemouth Cichlids) as previously described (27). Briefly, keratocytes were detergent extracted and the actin network was stabilized with phalloidin (50 nM Alexa-488 phalloidin (ThermoFisher) with 200 nM unlabeled phalloidin (MilliporeSigma)). The protocol for labeling protein with oligos and preparing the 6-site DNA origami is described above. Myosin VI was added in the final mix at a saturating concentration of 1 μM along with an ATP regenerating mix (1 mM phosphocreatine and 0.1 mg/ml creatine phosphokinase), an oxygen-scavenging system (0.6% glucose and 45 μg/ml catalase, 25 μg/ml glucose oxidase), and 2 mM ATP. Movies of adaptor–myosin VI scaffold motility on keratocyte were acquired at 1 Hz for 5 min per field of view. Trajectories from Trackmate (55) were imported into Matlab (Mathworks) using the @msdanalyzer package (60). Custom Matlab code was used to analyze trajectories for pause behavior. Briefly, tracks were filtered for trajectories that were longer than 10 frames, had a minimum average rate of displacement of 0.05 μm/sec, and a total displacement of at least 1 um. Pauses were defined as a drop to below 0.1 μm movement over four frames (4 s).

### Actin remodeling assay on supported lipid bilayers

Glass coverslips (22 × 22 mm, Corning) were soaked overnight in acetone and dried under a stream of nitrogen gas. Coverslips were sonicated in a bath sonicator for 30 min in a solution of 7× cleaning solution (MP Biomedicals) diluted sevenfold. Cleaning solution was moved by continuous washing with a stream of distilled water for 5 to 10 min. Coverslips were then bath sonicated again in 70% ethanol for 30 min. In total, 70% ethanol was replaced with 100% ethanol and bath sonication was repeated for an additional 30 min. Coverslips were stored in 100% ethanol for up to several days before use. Coverslips were dried under a stream of dry nitrogen before using double-sided adhesive tape to form flow chambers for supported lipid bilayer assembly. The lipid mixture was prepared by mixing 74.8 mol% phosphatidyl ethanolamine (PE), 25 mol% phosphatidylserine (PS), 0.1 mol% DGS-PE-biotin, and 0.1 mol% Cy5-PE in chloroform. The lipid mixture was dried by evaporating chloroform under a stream of nitrogen gas and then placed in a vacuum desiccator for 30 min. The dried lipid mixture was rehydrated by adding 1 ml of AB and mixed by pipetting and gentle vortexing. Liposome formation was carried out by incubating the rehydrated lipid mixture at 37 °C for 2 h. The liposome mix was then sonicated in a bath sonicator for 15 min to prepare a homogeneous preparation of liposomes. The liposome mix was then added to the flow chamber and incubated for 2 h to allow for supported lipid bilayer assembly on the glass coverslip surface. Postincubation, the flow chamber was washed with 200 μl of AB to remove any residual liposomes. Neutravidin at 0.2 mg/ml in AB.BSA was added to the flow chamber and incubated for 4 min. Biotinylated F-actin labeled with Alexa-647 phalloidin (1:9 ratio of biotin-G-actin: G-actin) was then added to the flow chamber at a concentration of 0.5 μM and incubated for 4 min to form an actin network on supported lipid bilayers. Myosin VI FL, myosin VI HMM dimer, and myosin V were added at a concentration of 200 nM in AB.BSA.Cam supplemented with an ATP regenerating mix (1 mM phosphocreatine and 0.1 mg/ml creatine phosphokinase), an oxygen-scavenging system (0.6% glucose and 45 μg/ml catalase, 25 μg/ml glucose oxidase), and 2 mM ATP. Similarly, ADP and AMP-PNP conditions contained 2 mM of the respective nucleotides instead of ATP. Dab2-MIR was added at a saturating concentration of 2 μM in the final mix. The actin network remodeling was imaged using a Nikon Eclipse Ti inverted epifluorescence microscope at 100× using a 1.4 NA oil-immersion objective at a frame rate of 1 Hz for 5 min using Nikon Elements software (Nikon). For the dual-color imaging of actomyosin, the imaging was carried out on a Zeiss TIRF microscope equipped with a Coherent 100 mW 488 nm and 561 nm OPSL laser and a 100× 1.4 NA oil-immersion objective with a DualView 2 for simultaneous two-channel imaging and a Photometrics QuantEM 512SC EMCCD camera. Data was captured using ZEN imaging software (Zeiss). Images were analyzed and prepared with FIJI (56, 57)

## Data availability

All data is contained within the article.

## Conflict of interests

The authors declare that they have no conflicts of interest with the contents of this article.
