# Supplementary material for: Dynamic multimerization of Dab2–Myosin VI complexes regulates cargo processivity while minimizing cortical actin reorganization
Source: J Biol Chem. 2021 Jan 7;296:100232. doi: 10.1074/jbc.RA120.012703 (PMC7948593; doi:10.1074/jbc.RA120.012703)
Supplement: Figure S1 [file mmc8.pdf]

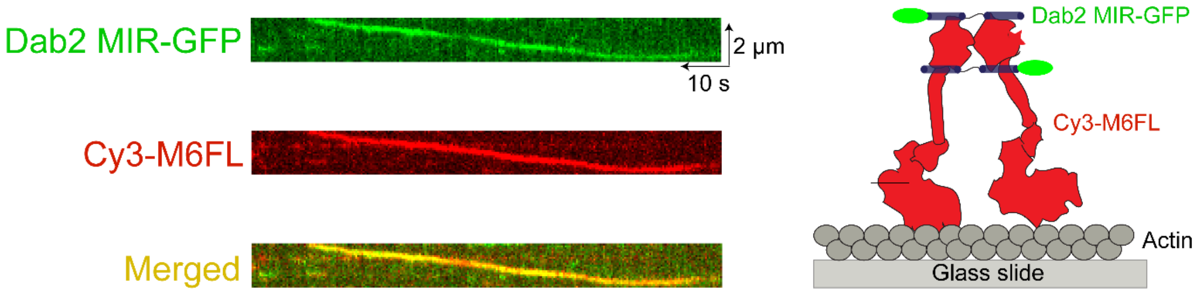


**Supplemental Figure 1: Dab2 MIR and myosin VI FL form a complex during single molecule processive motility.** Kymographs of a single Dab2 MIR-myosin VI FL complex during a processive run. Shown alongside is a schematic representation of the two-color single-molecule TIRF motility assay used to generate the kymographs.

**SUPPLEMENTAL MOVIE LEGENDS**

Movie S1-S3: Dual color single-molecule motility movies for the same spot shown as Dab2 MIR-GFP channel (Movie S5), Cy3-Myosin VI FL channel (Movie S6) and merge of the two channels (Movie S7)

Movie S4: Actin remodeling on supported lipid bilayers by ΔCBD dimer of myosin VI.

Movie S5: Actin remodeling on supported lipid bilayers by Dab2 + full-length myosin VI.

Movie S6: Merged dual color Alexa 488-actin (green) and Cy3-myosin VI (red) channels of a single aster during actin remodeling by ΔCBD dimer.

Movie S7: Merged dual color Alexa 488-actin (green) and Cy3-myosin VI (red) channels of a field of view of actin remodeling by ΔCBD monomer.
